# Learning a Filtered Backprojection Reconstruction Method for Photoacoustic Computed Tomography With Hemispherical Measurement Geometries

**DOI:** 10.1109/TMI.2025.3591706

**Published:** 2026-01

**Authors:** Panpan Chen, Seonyeong Park, Refik Mert Cam, Hsuan-Kai Huang, Alexander A. Oraevsky, Umberto Villa, Mark A. Anastasio

**Affiliations:** Department of Bioengineering, University of Illinois at Urbana–Champaign, Urbana, IL 61801 USA; Department of Bioengineering, University of Illinois at Urbana–Champaign, Urbana, IL 61801 USA; Department of Electrical and Computer Engineering, University of Illinois at Urbana–Champaign, Urbana, IL 61801 USA; Department of Electrical and Computer Engineering, University of Illinois at Urbana–Champaign, Urbana, IL 61801 USA; TomoWave Laboratories, Inc., Houston, TX 77054 USA; Oden Institute for Computational Engineering and Sciences and the Department of Biomedical Engineering, The University of Texas at Austin, Austin, TX 78712 USA; Department of Bioengineering, University of Illinois at Urbana–Champaign, Urbana, IL 61801 USA

**Keywords:** Photoacoustic computed tomography, optoacoustic tomography, image reconstruction

## Abstract

In certain three-dimensional (3D) applications of photoacoustic computed tomography (PACT), including *in vivo* breast imaging, hemispherical measurement apertures that enclose the object within their convex hull are employed for data acquisition. Data acquired with such measurement geometries are referred to as *half-scan* data, as only half of a complete spherical measurement aperture is employed. Although previous studies have shown that half-scan data can uniquely and stably reconstruct the sought-after object, no associated closed-form reconstruction formula has been reported. To accurately reconstruct images from half-scan data, optimization-based iterative reconstruction methods can be employed; however, they are computationally expensive. To address this limitation, a learning-based filtered backprojection (FBP) reconstruction method, referred to as the half-scan FBP method, is developed in this work. Because the explicit form of the filtering operation in the half-scan FBP method is not currently known, a learning-based method is proposed to approximate it. The proposed method is systematically investigated by use of virtual imaging studies of 3D breast PACT that employ ensembles of numerical breast phantoms and a physics-based model of the data acquisition process. The method is subsequently applied to experimental data acquired in an *in vivo* breast PACT study. The results confirm that the half-scan FBP method can accurately reconstruct 3D images from half-scan data, while offering a substantial computational speed-up over iterative methods. Importantly, because the sought-after inverse mapping is well-posed, the reconstruction method remains accurate even when applied to data that differ considerably from those employed to learn the filtering operation.

## I. Introduction

PHOTOACOUSTIC computed tomography (PACT), also known as optoacoustic tomography, is a rapidly developing imaging modality with great potential for a wide range of biomedical applications [1], [2], [3]. In PACT, a short laser pulse irradiates the object, leading to the absorption of optical energy [4]. This absorbed energy induces a localized rise in acoustic pressure due to the photoacoustic effect [5]. The resulting acoustic waves travel through the object and surrounding medium and are subsequently measured by ultrasonic transducers. From these measurements, the initial pressure distribution can be estimated by use of a tomographic reconstruction method [6], [7], [8]. PACT provides functional information with high optical contrast in the near-infrared wavelength range, where hemoglobin is one of the major endogenous optical molecular chromophores. It also achieves relatively high resolution, since the photoacoustic signals propagate as acoustic waves, which experience significantly less scattering than light by several orders of magnitude [9], [10], [11]. Such capabilities enable PACT to provide detailed structural and functional information about the distributions of oxy- and deoxy-hemoglobin concentration in tissues, making it a promising technique for cancer diagnosis and early screening by assessing tumor angiogenesis and hypoxia [12], [13], [14].

A variety of analytic reconstruction methods have been proposed for three-dimensional (3D) PACT [15], [16], [17], [18], [19], [20], [21]. Many of these methods employ filtered backprojection (FBP)-type approaches, where reconstruction is facilitated by first filtering measurement data and subsequently backprojecting this filtered data [15], [20], [21]. The derivations of FBP-based inversion formulae assume that photoacoustic signals are measured over certain canonical closed surfaces that enclose the object [15], [20], [21]. In certain modalities, such as 3D PACT breast imaging, physical constraints limit the measurement aperture for data acquisition. For example, in clinical breast imaging applications, hemispherical measurement surfaces [10] are employed. When standard FBP methods are directly applied to measurement data acquired with hemispherical measurement geometries, the resulting reconstructed images can exhibit inaccuracies and noticeable artifacts [22]. Hereafter, the data acquired with such a hemispherical measurement geometry will be referred to as *half-scan* data, with the assumption that the to-be-imaged object resides within the convex hull of the measurement aperture. Similarly, data acquired over a spherical surface that encloses the object will be referred to as *full-scan* data.

Previous studies have shown that half-scan data uniquely specify the sought-after object [23], [24], [25]. Moreover, results based on microlocal analysis [26], [27], [28] have established that half-scan data are sufficient for stable image reconstruction. Despite this, direct closed-form methods specifically designed for 3D image reconstruction from half-scan data are currently unavailable. Existing reconstruction methods for half-scan data are either heuristic modifications of direct methods that only partially mitigate artifacts [29], or computationally intensive iterative algorithms [23], [30]. Therefore, there remains a need for the development of accurate, direct 3D reconstruction methods for use with half-scan data.

To address this, a learning-based FBP-type reconstruction method, referred to as a half-scan FBP method, is proposed in this study. Because the explicit form of the filtering operation in the half-scan FBP method is not currently known, a learning-based method is proposed to approximate it, while offering a substantial computational speed-up over iterative methods. Key features of the method are that it seeks to approximate a well-posed inverse mapping and involves imaging physics in its formulation. As such, unlike the use of deep learning (DL)-based methods for solving ill-posed problems [31], the proposed method is expected to perform robustly even when applied to data that differ considerably from those employed for learning the filtering operation [32], [33], [34]. The accuracy of the method is systematically assessed through virtual imaging studies of 3D breast PACT that employ ensembles of numerical breast phantoms and a physics-based model of the data acquisition process. The method is subsequently applied to experimental data from an *in vivo* breast PACT study to further demonstrate its effectiveness under real-world conditions.

The remainder of the paper is organized as follows. In Section II, the PACT imaging model in its continuous and discrete forms is reviewed. The proposed half-scan FBP method is presented in Section III. The virtual imaging studies performed to validate and investigate the method are described in Section IV, with the corresponding results provided in Section V. In Section VI, the method is further validated by use of clinical data from an *in vivo* PACT breast study. Finally, the paper concludes with a discussion in Section VII.

## II. Background

In this section, continuous-to-continuous (C-C) and discrete-to-discrete (D-D) imaging models for 3D PACT are reviewed, along with relevant image reconstruction methods.

### A. Continuous-to-Continuous Imaging Model

The canonical C-C PACT forward imaging operator maps the sought-after initial pressure distribution $p_{0}(r)$, where $r\in R^{3}$, to the acoustic signals $p\left(r_{0},t\right)$ that are acquired over a measurement aperture $\Omega_{0}\subset R^{3}$, where $r_{0}\in \Omega_{0}$. Here, t∈[0,T] is the temporal coordinate confined to the acquisition period T, with tissue excitation assumed to occur at t=0. Assuming that the to-be-imaged object is acoustically homogeneous and lossless, with acoustic properties matching those of the acoustic coupling medium, the solution of the wave equation can be expressed by an integral equation [35]:

(1) $$\begin{array}{ll} p\left(r_{0},t\right) & =\frac{1}{4\pi c_{0}^{2}}\frac{d}{dt}\int_{V}dr\;p_{0}(r)\frac{\delta \left(t\;-\;\frac{\left|r_{0}-r\right|}{c_{0}}\right)}{\left|r_{0}\;-\;r\right|}\\ & ={ℋ}_{CC}\;p_{0}, \end{array}$$

where ${ℋ}_{CC}\;:L_{2}(R^{3})\to L_{2}\left(\Omega_{0}\times [0,T]\right)$ denotes the C-C imaging operator, δ(t) represents the one-dimensional Dirac delta function, $c_{0}$ is the constant speed of sound (SOS), and V denotes the object support.

### B. Discrete-to-Discrete Imaging Model

Consider that $N_{t}$ temporal samples of the pressure signal are recorded with a sampling interval of Δt at each of $N_{q}$ transducer locations on a hemispherical measurement surface that surrounds the to-be-imaged object. Because this work employs an FBP method, it is assumed that the density of transducer locations and temporal sampling frequency are sufficient [36] to avoid significant aliasing artifacts in the reconstructed images. The measured data samples can be lexicographically ordered and represented as the vector $p\in R^{M\times 1}$, where $M=N_{q}N_{t}$. Assuming idealized point-like transducers, the measured signal ${\left[p\right]}_{qN_{t}+w}$, recorded at time t=wΔt by the q-th transducer, is expressed as the $\left(qN_{t}+w\right)$-th element of the vector p:

(2) $${\left[p\right]}_{qN_{t}+w}={\left.p\left(r_{0},t\right)\right|}_{r_{0}=r_{q},t=w\Delta t},\;\begin{array}{rr} & w=0,\ldots ,N_{t}-1\\ & q=0,\ldots ,N_{q}-1 \end{array}.$$

To establish a D-D imaging model, the continuous object $p_{0}(r)$ is approximated by use of a finite-dimensional representation:

(3) $$p_{0}(r)=\sum_{n=0}^{N-1}{\left[f\right]}_{n}\phi_{n}(r),$$

where $[f{]}_{n}$ is the n-th element of the coefficient vector $f\in R^{N}$, and ${\left\{\phi_{n}(r)\right\}}_{n=0}^{N-1}$ is a set of given expansion functions. The D-D imaging model that maps the object coefficient vector f to the recorded data samples p can be expressed as [8]:

(4) p=Hf,

where $H\;:R^{N}\to R^{M}$ is the PACT D-D imaging operator, also referred to as the system matrix. In this study, an interpolation-based D-D imaging model was employed, with the expansion function chosen as piecewise linear basis functions [35]. As such, the coefficient vector f can be interpreted as a discretized approximation of the initial pressure distribution. Details regarding the implementation of H, including its GPU-parallelized formulation enabling on-the-fly computation, are available in [35].

### C. Image Reconstruction From Half-Scan Data

**Fig. 1.**
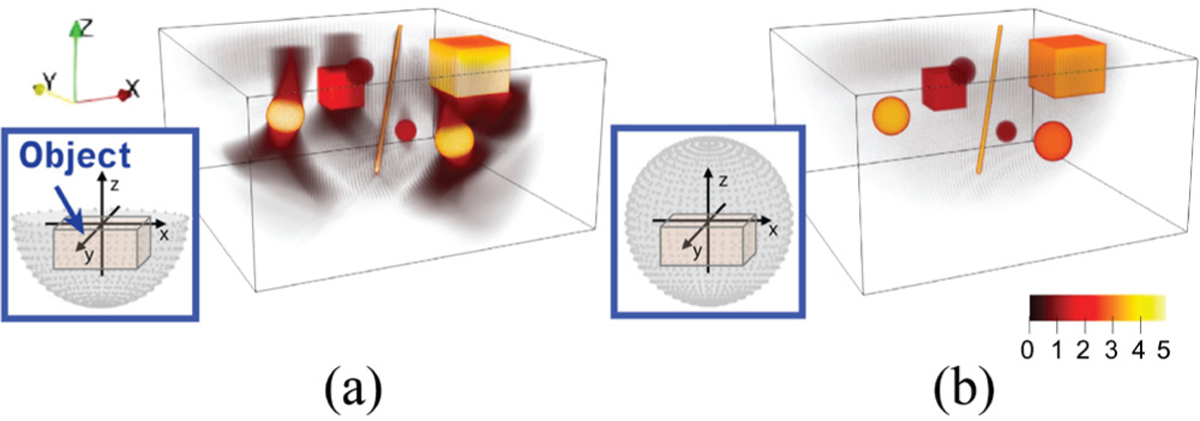
Standard FBP [38] results from (a) half-scan data and (b) full-scan data. Insets in (a) and (b) show the measurement geometries used for each data acquisition. In (a), direct application of the standard FBP method to half-scan data results in concentric arc-shaped artifacts originating from the endpoints of the open measurement surface boundary.

The direct application of analytic methods designed for full-scan data to reconstruct images from half-scan data can result in arc-shaped artifacts and loss of accuracy [28]. These artifacts appear as concentric arcs, centered at the endpoints of the open measurement surface and tangent to the object boundary [37]. As illustrated by the example in Fig. 1 (a), these arc-shaped artifacts are present in the image reconstructed from half-scan data using the standard FBP method, where the object boundaries parallel to the plane formed by the endpoints of the open measurement surface (the x-y plane at z=0) appear blurred.

Despite the fact that closed-form exact reconstruction formulae for use with half-scan data have not been identified, mathematical analyses on the invertibility of the C-C imaging model have yielded encouraging results. Specifically, it has been established that an object can be uniquely [23], [30] and stably [26] reconstructed from half-scan data. These findings established that the associated inverse problem in an infinite-dimensional vector space setting is well-posed. Iterative reconstruction methods can accurately reconstruct finite-dimensional object estimates from discrete samples of half-scan data [22], [23], [30]. However, iterative methods can be computationally expensive for 3D problems. As such, there remains an important need for the development of computational efficient and accurate reconstruction methods for use with half-scan data.

## III. Learned Half-Scan FBP Method

In this section, a semi-analytic discrete FBP-type method for use with half-scan data is developed. The method, referred to as the *learned half-scan FBP method*, is imaging physics-informed and designed to ensure accurate and rapid image reconstruction from half-scan data.

### A. Formulation of a Half-Scan Discrete FBP Method

The discrete form of a half-scan FBP-type reconstruction method can be expressed as

(5) $$\overset{ˆ}{f}=H^{†}Fp,$$

where $\overset{ˆ}{f}\in R^{N}$ denotes the reconstructed estimate of the object (a discrete approximation of the initial pressure distribution), $H^{†}\in R^{N\times M}$ is the adjoint of the D-D imaging operator, p is the measured half-scan data, and $F\in R^{M\times M}$ is the unknown data filtering operator. From the singular value decomposition (SVD) of H [33], a mathematical expression for the optimal linear filtering operator $F^{\mathrm{opt}}\in R^{M\times M}$ can be derived. However, computing the SVD of the large-scale imaging operator H in three dimensions is computationally challenging [39]. As a more practical alternative, the optimal filtering operator can be approximated by use of a supervised learning approach [33] as described next.

### B. Overview of the Learned Half-Scan FBP Method

In the proposed method, the optimal data filtering operator $F^{\mathrm{opt}}$ discussed above is approximated by use of a deep convolutional neural network, which is linear with respect to the measured half-scan data p. The convolutional neural network implements a data-to-data mapping that modifies the half-scan data p, serving as a spatially-variant and non-local filtering operation [40], [41]. This filtering network is denoted as $F_{\theta }\;:R^{M}\to R^{M}$, where the vector θ denotes the trainable network parameters. In this case, the task of estimating the optimal filtering operator can be interpreted as determining the parameter vector θ from a set of training data. Here, the training data consist of arbitrary pairs of objects $f^{\left(k\right)}$ and the corresponding half-scan measurement data $p^{\left(k\right)}\equiv {Hf}^{\left(k\right)}$, where the integer k is an index specifying each training sample. According to (5), the optimal parameter vector $\overset{ˆ}{\theta }$ should satisfy $f^{\left(k\right)}=H^{†}F_{\overset{ˆ}{\theta }}p^{\left(k\right)},\forall k$.

Therefore, to train the data filtering network $F_{\theta }$, the following optimization problem is approximately solved:

(6) $$\overset{ˆ}{\theta }=\underset{\theta }{argmin}\frac{1}{K}\sum_{k=1}^{K}ℒ(f^{\left(k\right)},H^{†}F_{\theta }p^{\left(k\right)}),$$

where K represents the number of training samples. The quantity ℒ(⋅,⋅) is the loss function, defined as the mean squared error (MSE) between the k-th reconstruction result ${\overset{ˆ}{f}}^{\left(k\right)}=H^{†}F_{\theta }p^{\left(k\right)}$ and the corresponding true object $f^{\left(k\right)}$.

**Fig. 2.**
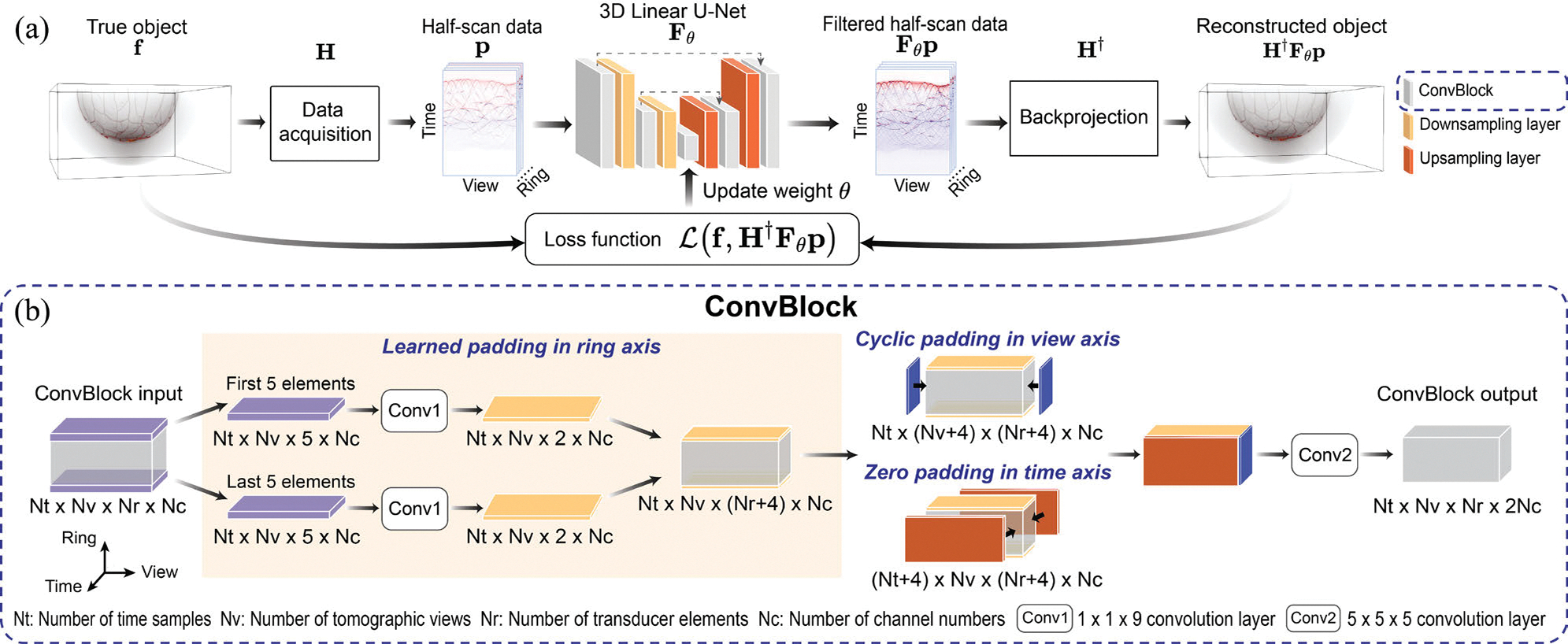
Learned half-scan FBP framework (a), where a linear 3D U-Net filters the half-scan data. This U-Net consists of convolution blocks (ConvBlocks) as shown in (b), with skip connections between corresponding encoder and decoder stages, along with downsampling and upsampling layers. The filtered data are subsequently backprojected to the image space for reconstruction. As illustrated in (b), each ConvBlock incorporates not only a convolution operation (Conv2) for feature extraction but also a padding approach based on prior knowledge of half-scan data, utilizing another convolution operation (Conv1) for learned padding.

Once the data filtering network is trained, it can replace F in (5) to form a learned half-scan FBP method, as depicted in Fig. 2 (a). Because of the well-posed nature of the inverse problem and the incorporation of imaging physics through $H^{†}$, the method is expected to perform well even when applied to measurement data that significantly differ from those employed for the filtering network training. In contrast, image-to-image-based DL approaches for image reconstruction rely on learning features in the image domain, which can limit their ability to generalize beyond the training distribution. As a result, such methods are more susceptible to generating inaccurate or misleading reconstruction, including false structures, when applied to unseen data [33], [42]. Appendix-A provides examples that support this conjecture.

### C. Network Architecture in the Learned Half-Scan FBP Framework

The following details regarding the assumed imaging system configuration, particularly the measurement geometry, are relevant to the specification of the data filtering network. Motivated by an existing 3D PACT breast imager [10], the system was assumed to comprise an arc-shaped acoustic measurement probe that was rotated in discrete steps to acquire data samples on a hemispherical measurement aperture. This configuration is parameterized by $N_{r}$, $N_{v}$, and $N_{t}$, which denote the number of transducer elements on the arc-shaped probe (ring dimension), the number of tomographic views (view dimension), and the number of time samples (time dimension), respectively.

Given that the half-scan data $p\in R^{N_{t}N_{q}\times 1}$, with $N_{q}=N_{v}N_{r}$, can be represented as a 3D tensor, a 3D U-Net model was employed to approximate the existing but unknown filtering operator. The 3D U-Net model was chosen as a canonical example of a deep neural network that has been widely deployed in medical imaging applications [43], [44]; however, alternative network models can also be employed for data filtering. Training was initially performed using an affine network architecture that excluded activation functions and max pooling but retained bias terms. During this training, the learned biases converged to values near zero, indicating that the affine network effectively approximated a linear mapping. Based on this observation, the network was subsequently fine-tuned adopting a strictly linear model by explicitly setting all bias terms to zero and fixing them throughout the remainder of the training. Appendix-A and Appendix-B present a comparative study of three architectural variants: a strictly linear model, an affine model, and a nonlinear model, along with the corresponding results.

The employed linear 3D U-Net consisted of encoder and decoder modules. The encoder was comprised of a series of convolution blocks (ConvBlocks) and downsampling layers, enabling the extraction of multi-scale feature representations. The downsampling layer was implemented using a 3D convolution with a kernel size of 3 × 3 × 3, a stride of 2, and an output channel size equal to the input channel size. As illustrated in Fig. 2 (b), each ConvBlock consisted of two convolution operations: one for learned padding in the ring dimension (Conv1), which will be detailed later, and another for feature extraction with a 5 × 5 × 5 kernel (Conv2). The encoder comprised of seven ConvBlocks, starting with 8 feature channels and successively doubling the number of channels at each block, culminating in 512 channels at the bottleneck ConvBlock. The decoder module, following the bottleneck, consisted of six ConvBlocks and upsampling layers. Transposed convolution was employed for upsampling, with a kernel size of 2 × 2 × 2, a stride of 2, and the same number of output channels as the input channels. The number of feature channels was halved after each upsampling step, and the resulting map was concatenated with the corresponding feature map from the encoder at the same level. Finally, a 1×1×1 convolution was applied to produce the single-channel U-Net output.

### D. Padding Approach for Half-Scan Data

To preserve the input dimensions through ConvBlocks, padding must be applied at the boundaries of the input data. Rather than using standard zero padding, the padding strategy in the ConvBlocks was designed based on the configuration of the assumed imaging system, as illustrated in Fig. 2 (b). For the view dimension, where tomographic views exhibited 2π periodicity, cyclic padding was employed to maintain azimuthal continuity by replicating values from one edge of the data to the opposite edge. In the time dimension, assuming temporally untruncated measurement, zero padding was applied by appending zeros to both ends of the temporal traces, as pressure traces outside the measured time range were considered negligible. Along the ring dimension, a learned padding approach was implemented, employing learned values to account for the unmeasured data beyond the transducer arc. This process began by extracting the first and last five elements of the input data along the ring axis, which were then processed through 1 × 1 × 9 convolution layers (Conv1). The outputs from Conv1 were subsequently concatenated with the original input data along the ring axis.

To assess the effectiveness of this physically informed padding approach, a comparative study was conducted. The results, provided in Appendix-B, show that the proposed physics informed padding approach achieved performance similar to the standard padding approach. However, the proposed approach better reflects the data acquisition process and was employed in the presented studies.

## IV. Virtual Imaging Studies: Implementation and Setup

This section outlines the virtual imaging studies conducted to validate the learned half-scan FBP method. These studies assess the method’s accuracy on a dataset whose characteristics match the training data, hereafter referred to as the in-distribution (ID) test set, and its generalizability on datasets that differ from the training data, referred to as out-of-distribution (OOD) test sets. To generate these datasets, two types of to-be-imaged objects were utilized: stochastically generated anatomically realistic 3D numerical breast phantoms (NBPs) [45] and 3D mouse phantoms [46], both incorporating realistic functional and optical properties. Six datasets were produced, varying by imaging system configurations and noise considerations. One of them was employed as the ID dataset, and the remaining five were used as OOD test sets. Further details are provided below.

### A. Virtual PACT Imaging and Data Acquisition

#### 1) Virtual Imaging System Configuration:

Three different virtual 3D PACT imaging systems were configured to emulate real-world imaging scenarios, each differing in their optical illumination subsystem design. The first system, referred to as *System A*, employed 20 evenly positioned arc-shaped illuminators (radius of 145 mm, central angle of 80°), each equipped with five linear fiber-optic segments along the arc-shaped surface. Each segment was represented by a broadening slit light source (half-angle of 12.5°). The second system, *System B*, used a single cone beam (half angle of 36°) to illuminate the object from the bottom upwards, while the third system, *System C*, applied uniform illumination across the object surface. All three systems employed an identical acoustic measurement subsystem. Acoustic signals were detected by 107 idealized point-like transducer elements uniformly distributed along the surface of an arc-shaped photoacoustic probe (radius of 85 mm, central angle of 90°), with an angle of approximately 0.84° between adjacent elements. The probe was rotated to form a complete hemispherical aperture, collecting data across 320 tomographic views during scanning. The number of measured time samples was set to 1280, with a sampling frequency of 10 MHz.

#### 2) Simulation of the Photoacoustic Effect:

To establish the 3D initial pressure distributions representing the to-be-imaged objects in the acoustic simulation, the optical energy deposition in the numerical phantoms was simulated using the GPU-accelerated MCX software [47] for Systems A and B, and the diffusion approximation [48] for System C. In the virtual imaging Systems A and B, all fiber-optic segments and the single cone beam were simulated using 10^10^ and 5×10^10^ photons, respectively, both over 50 ns. An illumination wavelength was randomly selected from 757 nm, 800 nm, and 850 nm for each object in both systems. Virtual imaging System C involved uniform illumination on the object surface at a wavelength of 800 nm.

#### 3) Simulation of Acoustic Measurement Acquisition:

Acoustic measurements were simulated by use of the interpolation-model-based D-D forward model described by (4). A reference SOS of 1509.15 m/s was assumed, which was chosen based on experimental breast data used in the *in vivo* study presented in Section VI. Full-scan data were also simulated for use in reconstructing high-quality reference images. In the full-scan data acquisition, which is not physically realizable with *in vivo* breast PACT, a spherical aperture was formed by scanning with an arc-shaped probe (radius of 85 mm, central angle of 180°) equipped with 213 evenly distributed transducer elements, each spaced approximately 0.84° apart, and rotated in 320 discrete steps. Additionally, noisy versions of the measurements were produced. Additive noise was modeled as an independent and identically distributed Gaussian random variable with a mean of zero and a standard deviation equal to 1% of the maximum photoacoustic signal amplitude observed across all training, validation, and ID testing sets [49]. These datasets are detailed in the next subsection.

#### 4) Training, Validation, and Test Datasets:

**Fig. 3.**
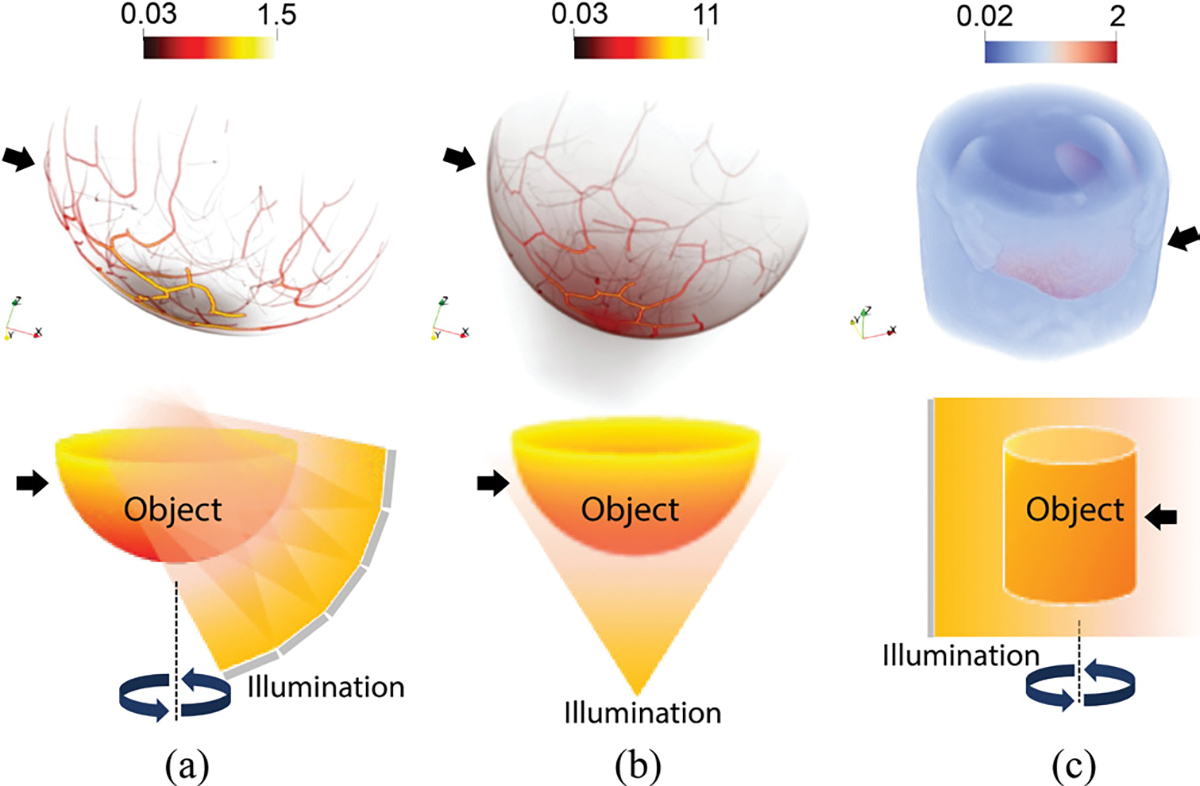
Examples of 3D initial pressure distributions (top) and the corresponding light delivery system configurations (bottom) for (a) NBP-A (b) NBP-B, and (c) MOBY datasets, used in virtual imaging Systems A, B, and C, respectively. Volume rendering was performed in Paraview [50], where intensities are accumulated along the viewing direction based on the chosen color and opacity maps.

Different ensembles of data were generated for training and testing the learned half-scan FBP method. Each ensemble comprised to-be-imaged objects (initial pressure distributions) and their associated simulated measurement data. Recall that the initial pressure distributions are determined by both the optical properties of the object as well as the design of the light delivery subsystem in the PACT imager. Figure 3 illustrates examples from the three classes of initial pressure distributions that were considered. The ensembles derived from NBPs using the virtual imaging System A setup in the absence and presence of measurement noise are referred to as the *noiseless NBP-A* and *noisy NBP-A* datasets. Similarly, those generated from NBPs with the virtual imaging System B setup constitute the *noiseless NBP-B* and *noisy NBP-B* datasets. The final ensembles, simulated using abdomen-region-cropped 3D numerical mouse whole-body (MOBY) phantoms [46] with the virtual imaging System C setup, are referred to as the *noiseless MOBY* and *noisy MOBY* datasets. These MOBY datasets were utilized because they exhibit anatomical structures that differ significantly from those seen in the training data [51], thereby enabling a challenging OOD test. Additionally, variations in illumination schemes across the systems were employed to generate objects with distinct modulations of the initial pressure distribution, even among subjects with similar anatomies. The acoustic measurement geometry remained fixed.

The learned half-scan FBP method was trained on the noiseless NBP-A dataset. To evaluate the method’s generalizability, five OOD test sets were employed. The noisy NBP-A dataset, which utilized the same type of to-be-imaged objects as the training data but under noisy conditions, served as one OOD test set. The noiseless and noisy NBP-B datasets, representing more challenging scenarios through varied illumination patterns, constituted the second type of OOD datasets. Finally, the noiseless and noisy MOBY datasets presented the most challenging OOD conditions by varying both the illumination patterns and the objects to be imaged. A total of 5000 noiseless NBP-A samples were divided into 4500 training, 250 validation, and 250 test samples. The OOD test sets consisted of 250 noisy NBP-A, 80 noiseless and noisy NBP-B, and 10 noiseless and noisy MOBY samples.

### B. Implementation of the Learned Half-Scan FBP

The implementation of the learned half-scan FBP method involves a 3D linear U-Net as the data filtering model and the backprojection operation $H^{†}\in R^{N\times M}$. Here, M was set to 1280 × 320 × 107, based on the total number of time samples, tomographic views, and transducer elements, as described in Section IV-A.1. The number of elements representing the discretized object N was set to 340 × 340 × 170, corresponding to a physical volume of 170 × 170 × 85 mm^3^ with a voxel size of 0.5 mm. This voxel size, along with the sampling frequency of 10 MHz in the acoustic measurement system, was chosen based on the available memory in the employed GPU. The interpolation-model-based D-D forward operation H and backprojection operation $H^{†}$ were implemented in C++ and accelerated using the CUDA library [35].

The 3D linear U-Net-based data filtering model $F_{\theta }$ was implemented in PyTorch [52]. To enable automatic differentiation of the loss function in (6), a user-defined PyTorch class was implemented to evaluate the actions of H and $H^{†}$. The Adam optimizer was utilized for training, with a learning rate set to 10^−4^ and a batch size of 1. To accelerate training, the loss function was calculated from partial object volumes sized 340 × 85 × 170 voxels, randomly selected along the y-axis. Additional comparative experiments, detailed in Appendix-B, demonstrate that training the network with a loss function computed over randomly selected partial volumes yields reconstruction accuracy comparable to that achieved with full-volume loss computation, while enabling approximately a 2× speed-up in training time.

The model was trained on a GPU compute node in the Delta advanced computing resource [53], utilizing 8 NVIDIA A100 GPUs with 40 GB of memory per GPU, and an AMD 64-core 2.45 GHz Milan processor. During training, a single GPU was designated for U-Net model training, while the remaining seven GPUs were utilized to parallelize the computations of D-D imaging models: $H^{†}$ for loss evaluation and H for the backpropagation process. The network was trained for 50 epochs over approximately 48 days, with each epoch averaging 23 hours, of which approximately 17 hours were required to compute the D-D models. Peak GPU memory usage during training reached approximately 39 GB.

The testing of the proposed method, as well as the implementation of other reference methods, was conducted on a system equipped with 4 NVIDIA A100 GPUs with 80 GB of memory per GPU and an Intel^®^ Xeon^®^ Silver 4309Y CPU @ 2.80GHz. To reconstruct one image from the test set using the proposed learned FBP method, similar to training, one GPU was designated for loading the trained U-Net model, which required approximately 29 GB of GPU memory, while the remaining three GPUs parallelized the computation of backprojection operator $H^{†}$. The proposed method took 30 seconds to reconstruct a single object, with the $H^{†}$ computation accounting for 28 seconds, while the learned filtering operation took only 2 seconds.

### C. Study Designs and Evaluations

The following studies were performed to assess the accuracy, stability, and generalizability of the learned half-scan FBP method. First, the method was established by training the data filtering network on the noiseless NBP-A training set, and its reconstruction performance was evaluated on the noiseless NBP-A test set. Without retraining, the method was then tested on five OOD test sets: the noisy NBP-A dataset, as well as the noiseless and noisy NBP-B and MOBY datasets. In all cases involving noisy data, a Gaussian low-pass filter with a half width half maximum (HWHM) of 0.1177*μ*s was implemented in SciPy [54] and applied to the simulated noisy measured data prior to image reconstruction. This is equivalent to apodizing the data filtering operation, which is a common approach to mitigating noise amplification in FBP-type methods [15].

The learned half-scan FBP method was qualitatively and quantitatively assessed through comparison with the standard FBP method applied to both half-scan and full-scan data. The same Gaussian low-pass filter mentioned above was applied as a data preprocessing step when employing the standard FBP method. Visual inspections were conducted through volume rendering and line profile comparisons. Reconstructed images were quantitatively assessed by use of MSE and the structural similarity index (SSIM), which were calculated with respect to the true objects. These assessments were performed using both ID and OOD testing datasets.

## V. Virtual Imaging Results

**Fig. 4.**
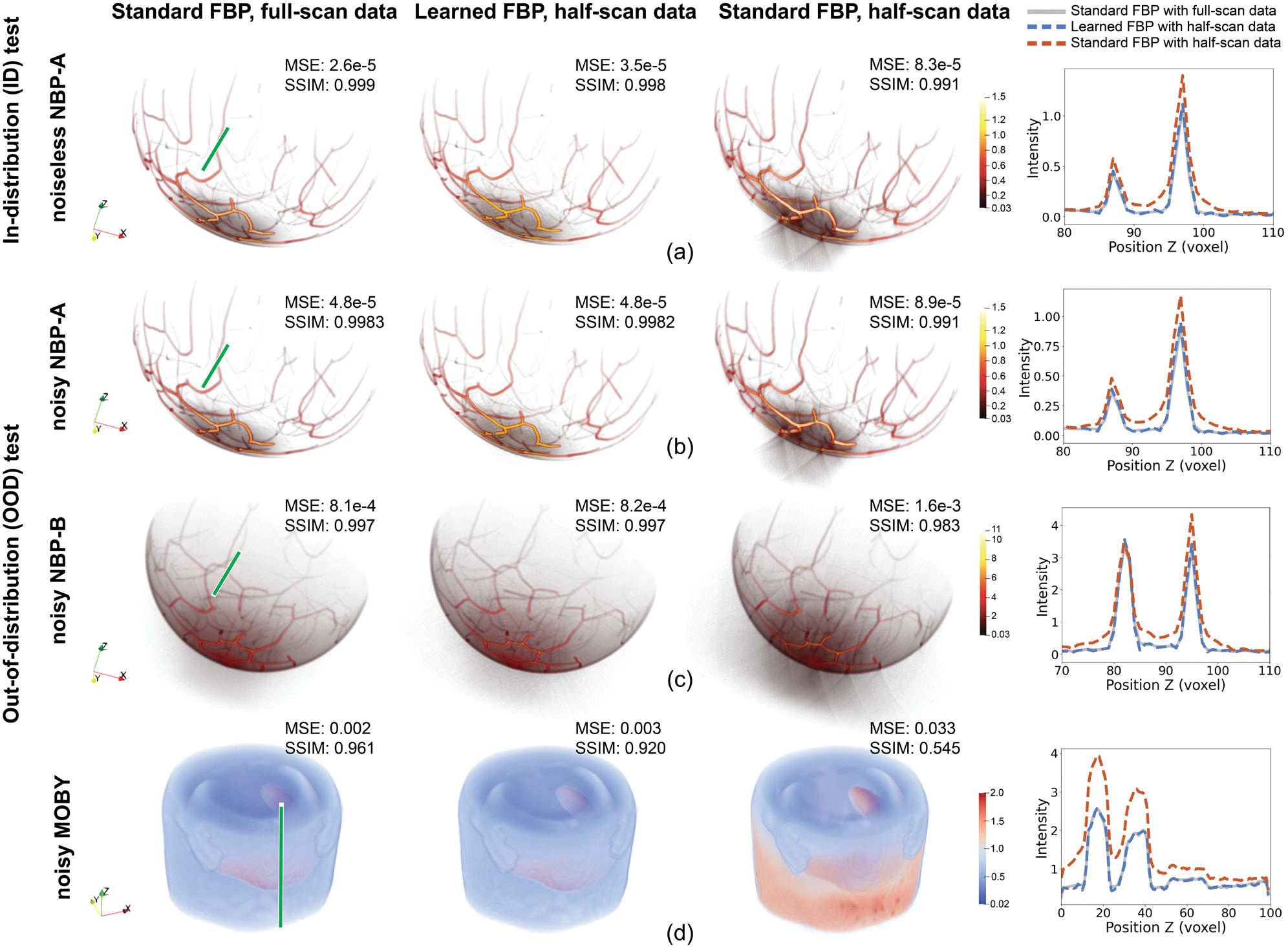
Visual inspections (first to third columns) and line profile comparisons (fourth column) for the ID test from (a) the noiseless NBP-A dataset and for the OOD tests from (b) the noisy NBP-A, (c) noisy NBP-B, and (d) noisy MOBY datasets. The first to third columns display the volumes reconstructed using the standard FBP method applied to full-scan data, the learned half-scan FBP method, and the standard FBP method applied to half-scan data, respectively. Volume rendering was performed using Paraview. The line along which the profiles compared in the rightmost column were extracted is annotated in the volumes contained in the first column, indicated by green lines. In the profile comparisons (fourth column), the gray solid line, blue dashed line, and red dashed lines correspond to the standard FBP method applied to full-scan data, the learned half-scan FBP method, and the standard FBP method applied to half-scan data, respectively. The comparisons reveal that the learned half-scan FBP method significantly outperformed the standard FBP method applied to half-scan data and performed comparably to the standard FBP method applied to full-scan data, for both ID and OOD test samples, even in the presence of noise.

Figures 4 (a) and (b) present examples of images reconstructed using the noiseless and noisy NBP-A data, which correspond to the ID and OOD test results, respectively. Both visual inspection and line profiles confirmed that the images reconstructed by use of the standard FBP method applied to full-scan data (left column) and the learned half-scan FBP method (second column) were nearly indistinguishable, which was consistent with the similar MSE and SSIM values reported in the figures. This confirmed that the proposed method can reconstruct images from half-scan data that closely approximate ones reconstructed using the standard FBP method applied to full-scan data, even in the presence of measurement noise. On the other hand, both the noiseless and noisy images reconstructed by use of the standard FBP method from half-scan data (third column) exhibited arc-shaped artifacts, consistent with the predictions of the microlocal analysis findings mentioned in Section II-B. These artifacts caused a widening of the object structures along the z-axis, as also revealed by the provided line profile comparisons. Additionally, the MSE and SSIM values for these images were found to be inferior to those corresponding to the learned half-scan FBP reconstructions.

Figures 4 (c) and (d) illustrate the OOD test results from noisy measurements simulated using the NBP-B and MOBY datasets, respectively. As in the ID testing results, the learned half-scan FBP method (second column) performed comparably to the standard FBP method applied to full-scan data (first column) and outperformed the standard FBP method applied to half-scan data (third column), substantially reducing artifacts. These conclusions are further corroborated by the line profile comparisons and the reported MSE and SSIM values.

**Fig. 5.**
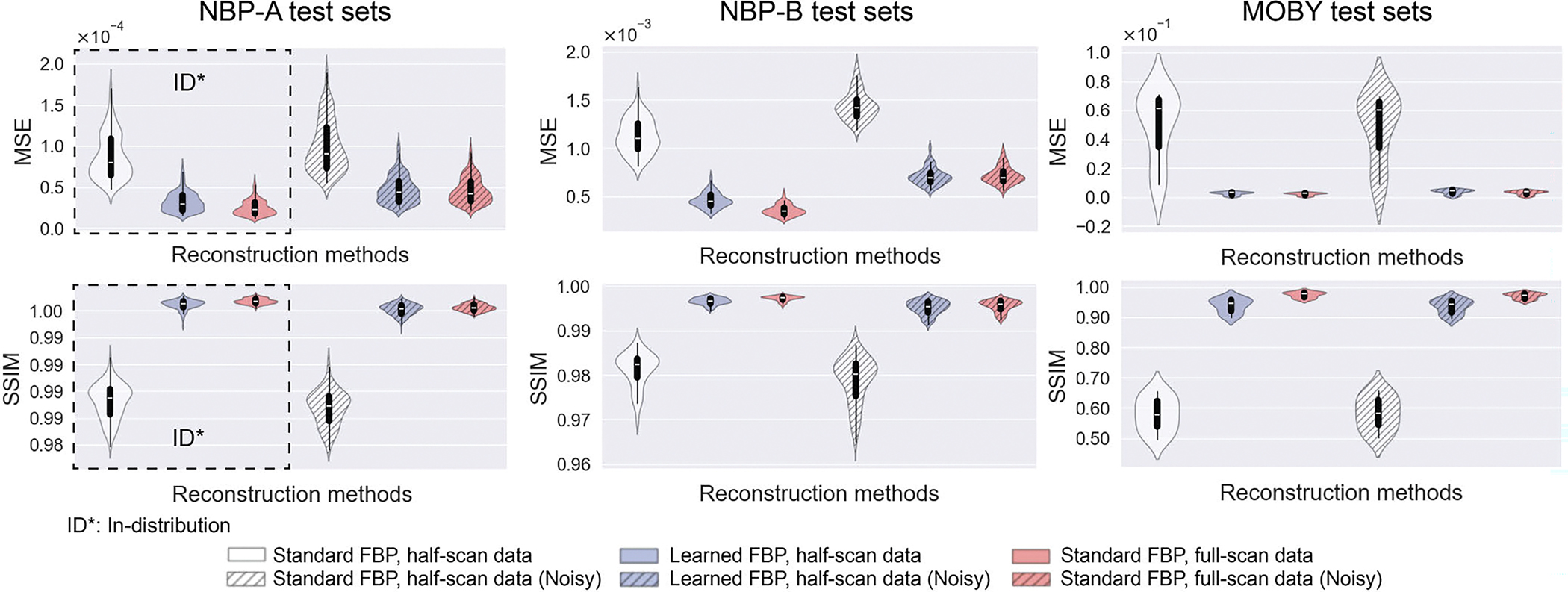
Violin plots of the MSE (top row) and SSIM (bottom row) values for the standard FBP method applied to half-scan data (white), the learned half-scan FBP method (blue), and the standard FBP method applied to full-scan data (red) under noiseless (solid fill) and noisy conditions (diagonally striped fill). Results are shown for 250 NBP-A (first column), 80 NBP-B (second column), and 10 MOBY samples (third column) for each method. The dashed box in the first column indicates the results from the ID test, while the remaining plots represent the OOD test results. The quantitative results indicate that the learned half-scan FBP method (blue) is robust in noisy conditions (diagonally striped fill) and exhibits strong generalizability across different types of objects (second and third columns), achieving a reconstruction accuracy comparable to the standard FBP method applied to full-scan data (red).

Figure 5 presents violin plots of the MSE and SSIM values for the standard FBP method applied to half-scan data (white), the learned half-scan FBP method (blue), and the standard FBP method applied to full-scan data (red), under noiseless (solid fill) and noisy conditions (diagonally striped fill). Results are shown for 250 NBP-A, 80 NBP-B, and 10 MOBY samples for each method. Consistent with the results in Fig. 4, the proposed method demonstrated robustness in noisy conditions and strong generalizability across different types of objects.

## VI. Application to Experimental Breast Data

The performance of the learned half-scan FBP method under real-world conditions was explored by use of experimental breast PACT data acquired in an *in vivo* study.

### A. Data Acquisition

In this study, archived, fully-anonymized experimental breast data [10] were utilized. These data were acquired from both the left and right breasts of a healthy female volunteer at an illumination wavelength of 755 nm. Data collection was performed using the LOUISA-3D system (TomoWave Laboratories, Houston, TX) [10] at MD Anderson Cancer Center. The LOUISA-3D system, whose acoustic detection system was modeled in the virtual imaging studies, was equipped with a photoacoustic probe (radius of 85 mm, center angle of 80°) containing 96 ultra-wideband (50 kHz to 6 MHz) transducer elements, each with a square shape sized 1.1 × 1.1 mm^2^ and spaced approximately 0.84° apart. By rotating the probe in discrete steps, a spherical segment aperture was formed. In this experimental study, 1536 pressure time samples were collected over 76.8 *μ*s at a sampling frequency of 20 MHz [10].

### B. Image Reconstruction and Evaluation

The learned half-scan FBP method that was trained on the noiseless NBP-A dataset in the virtual imaging studies was applied to *in vivo* data. To accommodate for differences in the data acquisition design between the LOUISA-3D system and the virtual imaging system used to train the filter, the following preprocessing steps were applied for the learned half-scan FBP method. First, a Gaussian low-pass filter with a HWHM of 0.05887 *μ*s was applied to the experimental data to mitigate noise. Next, the experimental data were subsampled at a sampling frequency of 10 MHz to match that used in the virtual imager. Finally, experimental data were zero-padded along the time and transducer ring dimensions to match the number of time samples and transducer rings expected by the pretrained filter $F_{\theta }$.

To evaluate the performance of the learned half-scan FBP method, two reference methods were considered: the standard FBP method applied to the acquired half-scan data and an iterative method in which a penalized least squares (PLS) problem with total variation (TV) regularization was solved using the Fast Iterative Shrinkage-Thresholding Algorithm, referred to as *FISTA-TV* [55]. The FISTA-TV reconstruction served as the reference estimate of the true object. Given the sufficiency of the half-scan data, such methods are known to yield accurate images from half-scan data [23], [56]. For the standard FBP method, the following preprocessing steps were applied. The experimental data were first smoothed with a Gaussian low-pass filter with a HWHM of 0.05887 *μ*s and then subsampled to a sampling frequency of 10 MHz. For the FISTA-TV method, no preprocessing was applied to the experimental data. The regularization parameter was set to 4×10^−3^ after a parameter sweep within the range [1×10^−4^, 1 × 10^−2^] and chosen based on visual examination, with a balance between noise suppression and the preservation of image details as the selection criterion. The algorithm terminated when ${\left\|f^{\left(i\right)}-f^{\left(i-1\right)}\right\|}^{2}/{max}_{l\leq i}{\left\|f^{\left(l\right)}-f^{\left(l-1\right)}\right\|}^{2}$ fell below a threshold of 5 × 10^−2^, where i and l denote the iteration index [57]. For image reconstruction of the left and right breasts, constant SOS values of 1509.15 m/s and 1503.00 m/s were subjectively selected, respectively.

### C. Experimental Results

**Fig. 6.**
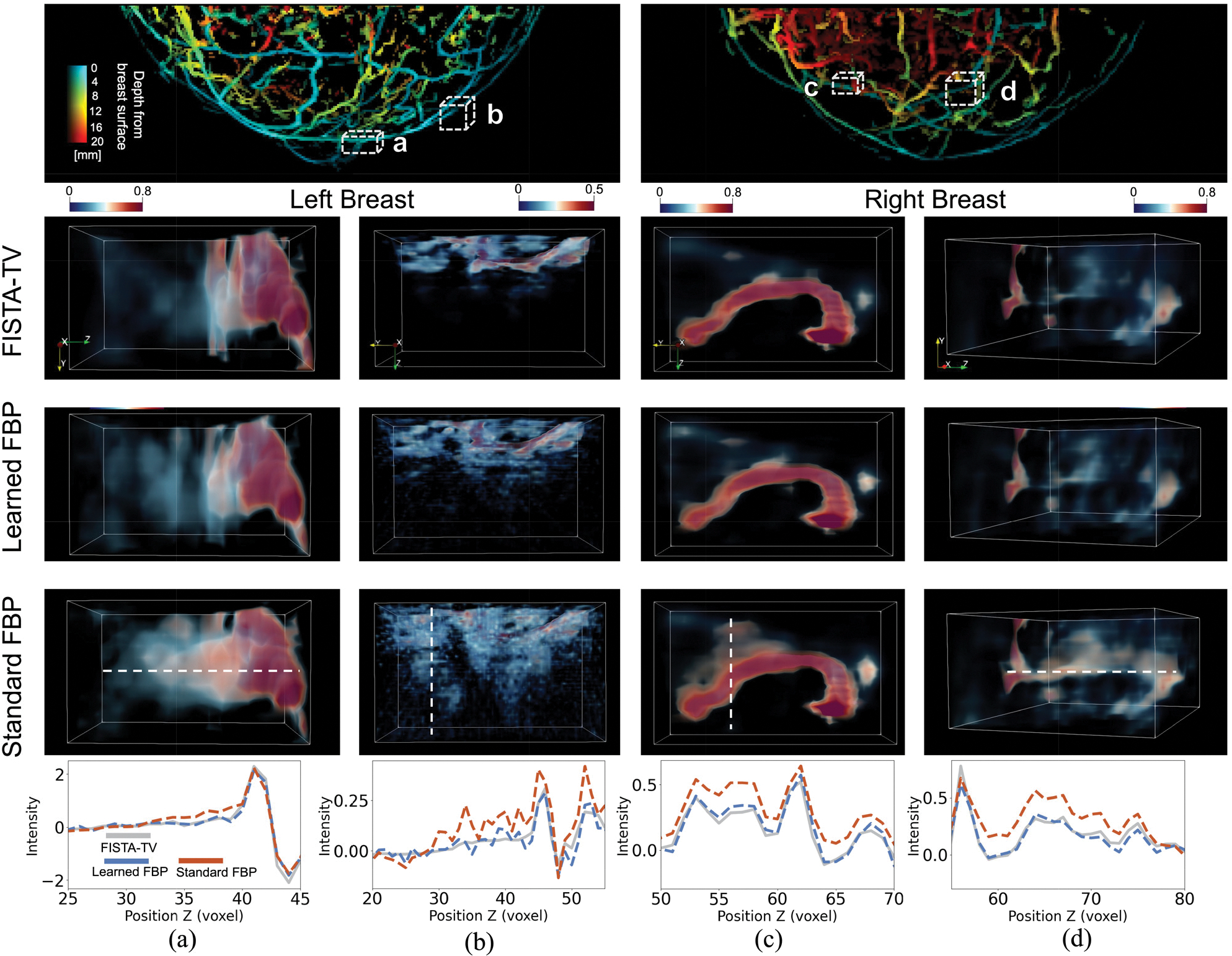
Visual inspection of images reconstructed from *in vivo* breast data using three methods: the FISTA-TV, learned half-scan FBP, and standard FBP methods. The top row, from left to right, displays images of the entire left (a and b) and right breasts (c and d), reconstructed using the learned half-scan FBP method with optical fluence normalization [49] and depth-based color coding applied. The second to fourth rows show zoomed-in regions of the images reconstructed by use of each method, respectively. The selected regions are highlighted by white boxes in the top row. White dashed lines in the zoomed-in regions in the fourth row indicate the locations of profiles compared in the bottom row. The results demonstrate that the learned half-scan FBP method generalizes effectively to experimental data, mitigating artifacts and inaccuracies observed when the standard FBP method is applied to half-scan data. A video demonstrating the complete 3D images reconstructed by the three methods is provided in the supplementary materials.

Figure 6 presents images that were reconstructed from experimental data. The top row of Fig. 6 shows images of the entire left and right breasts, from left to right, reconstructed using the learned half-scan FBP method. For display purposes, optical fluence normalization and depth-based color coding were applied [49]. These images also indicate the locations of zoomed-in regions for method comparisons ((a) and (b) for the left breast, (c) and (d) for the right breast), marked with dashed boxes. The second, third, and fourth rows display the zoomed-in regions reconstructed from half-scan data using the FISTA-TV method, the learned half-scan FBP method, and the standard FBP method, respectively. The bottom row shows line profiles from the zoomed-in regions, with white dashed lines in the fourth row indicating their locations. Compared to the reference images reconstructed by use of the FISTA-TV method, the image reconstructed using the standard FBP method from half-scan data exhibited artifacts and false connectivity in the object structure. In contrast, the proposed method effectively suppressed these artifacts, achieving relatively high reconstruction accuracy. The line profile comparison in the bottom row of Fig. 6 further substantiates these findings. A video demonstrating the complete 3D object estimates reconstructed using the FISTA-TV, learned half-scan FBP, and standard FBP methods is provided in the supplementary materials.

Table I provides the MSE and SSIM values of images reconstructed by use of the learned half-scan and standard FBP methods, relative to the images reconstructed using FISTA-TV that served as references. The average reconstruction times are also provided. The learned half-scan FBP method achieved performance comparable to the FISTA-TV method, while reducing reconstruction times by a factor of 1000. On the other hand, the standard FBP method yielded inferior MSE and SSIM values compared to the learned half-scan FBP method. It is noteworthy that out of the 30 seconds required by the learned half-scan FBP method, only 2 seconds were attributable to the U-Net-based data filtering. The remaining time was spent on the interpolation-model-based backprojection operation, which can vary depending on the implementation.

## VII. Discussion and Conclusion

Prior studies have established that PACT image reconstruction from data acquired with a hemispherical measurement geometry, i.e., half-scan data, corresponds to a well-posed problem. However, no closed-form inversion formula for this problem is currently available. To address this, a learned half-scan FBP method was proposed, in which a linear deep neural network approximates the unknown data filtering operation for half-scan data. The conducted studies confirmed that the learned half-scan FBP method achieved high reconstruction accuracy, comparable to the FISTA-TV reference method, while reducing reconstruction time by approximately three orders of magnitude. Importantly, the learned half-scan FBP method exhibited robust generalizability, even when applied to experimental breast data acquired in an *in vivo* study. Because of these characteristics, the learned FBP method may benefit applications of PACT that include structural and functional breast imaging.

The robust generalizability of the proposed method stems from the fact that inverse problem associated with PACT image reconstruction from half-scan data is well-posed. A recent work by Cam et al. [33] employed a similar strategy, in which a learned FBP method was established for approximately inverting the so-called half-time circular Radon transform. Perhaps the first study that proposed a learned FBP method for a well-posed problem was reported by Floyd [40]. In such applications, the instabilities commonly observed in DL-based image reconstruction approaches [58] are avoided, resulting in reliable and trustworthy methods. In addition, learning-based FBP methods have been investigated not only by replacing the entire FBP pipeline with a neural network [41], but also through modular designs [59] in which individual components, such as filtering and backprojection, are modeled as distinct, learnable modules [60], [61].

In the virtual imaging studies where full-scan data were available, small discrepancies in MSE and SSIM values were observed between the learned half-scan FBP method and the standard FBP method applied with full-scan data, as shown in Fig. 5. These discrepancies may have resulted from the fact that, having a learned filtering component, the proposed method only approximates the true inverse mapping. Furthermore, full-scan data contain redundancies, whereas half-scan data do not [23], which will result in numerical errors and noise being propagated differently into image space.

The half-scan data for network training were simulated with a reference SOS of 1509.15 m/s, chosen empirically. To confirm the trained network’s robustness across a range of SOS values possible in *in vivo* breast imaging, the MSE and SSIM values for the learned half-scan FBP method were evaluated within the reported range of breast tissue SOS values (1447 m/s to 1555 m/s) [62]. The resulting MSE values were 4.6×10^−5^±1.6×10^−6^ and SSIM values were 0.997±2×10^−4^, demonstrating the method’s robustness across this SOS range. Therefore, for soft tissue PACT imaging where a homogeneous SOS assumption is generally useful, the DL model can be trained with a reference SOS and is expected to maintain high reconstruction accuracy across the SOS range without the need to retrain the model.

The extensive training and testing times reported in this study are primarily attributed to the computationally intensive interpolation-model-based matched D-D imaging operators [35], H and $H^{†}$, which account for over 70% and 90% of the training and inference time, respectively. The use of this backprojection operator also explains the notably slower performance of the learned half-scan FBP method compared to the standard FBP method, as shown in Table I, which employs a more computationally efficient backprojection operator. Nevertheless, alternative implementations of the chosen D-D imaging model, the use of alternative D-D models with different expansion functions, or even the adoption of unmatched models [63], [64] may be explored to accelerate the overall training and inference processes.

Although this study was conducted with a specific PACT imager design, the proposed method can be readily adapted to other PACT imaging system designs. In particular, the proposed method is not restricted to hemispherical measurement geometries and is expected to yield stable reconstructions for any acoustic measurement geometry in which the object is fully enclosed by the convex hull of the measurement surface, provided that the measurements are sufficiently densely sampled [23], [26], [27], [28], [30]. In addition, any suitable DL model can be used for half-scan data filtering.

## Supplementary Material

supp1-3591706

This article has supplementary downloadable material available at https://doi.org/10.1109/TMI.2025.3591706, provided by the authors.

## Figures and Tables

**TABLE I:** MSE, SSIM, and Average Reconstruction Time

Method	MSE (Left, Right breast)	SSIM (Left, Right breast)	Average time
Standard FBP	5.3e-3, 1.1e-3	0.977, 0.973	3 s
Learned half-scan FBP	4.6e-3, 9.8e-4	0.985, 0.982	30 s
Backprojection model of Learned half-scan FBP	1.1e-2, 2.5e-3	0.953, 0.953	28 s

The FISTA-TV reconstruction took approximately 8 hours for each breast.

**Fig. 7.**
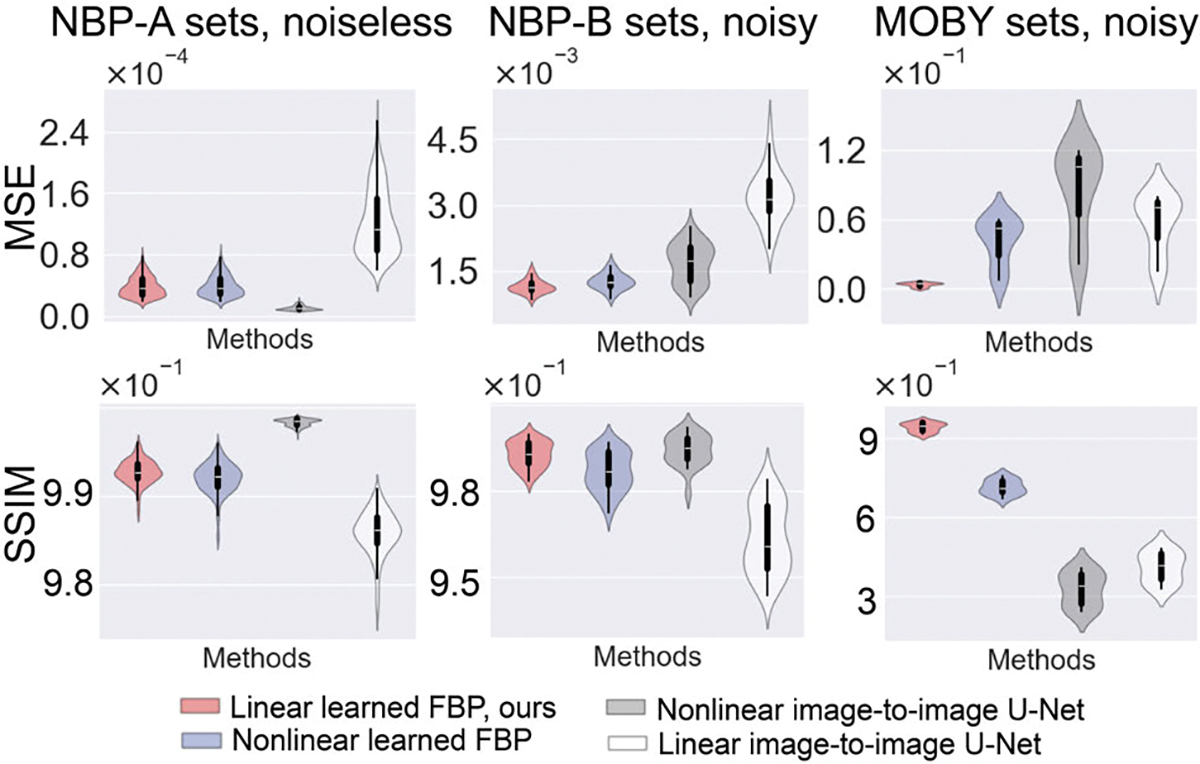
Violin plots of the MSE (top row) and SSIM (bottom row) values for the proposed linear learned half-scan FBP method (red), the nonlinear learned half-scan FBP method (blue), the linear image-to-image U-Net (white), and the nonlinear image-to-image U-Net (gray), on both ID (first column) and noisy OOD test sets (second and third columns). Results are presented for 250, 80, and 10 samples from the noiseless NBP-A (first column), noisy NBP-B (second column), and noisy MOBY datasets (third column), respectively. The linear learned half-scan FBP method (red) exhibits strong generalizability, significantly outperforming the other models, highlighting the effectiveness of data-domain filtering within the FBP framework, guided by the underlying imaging operator.

**Fig. 8.**
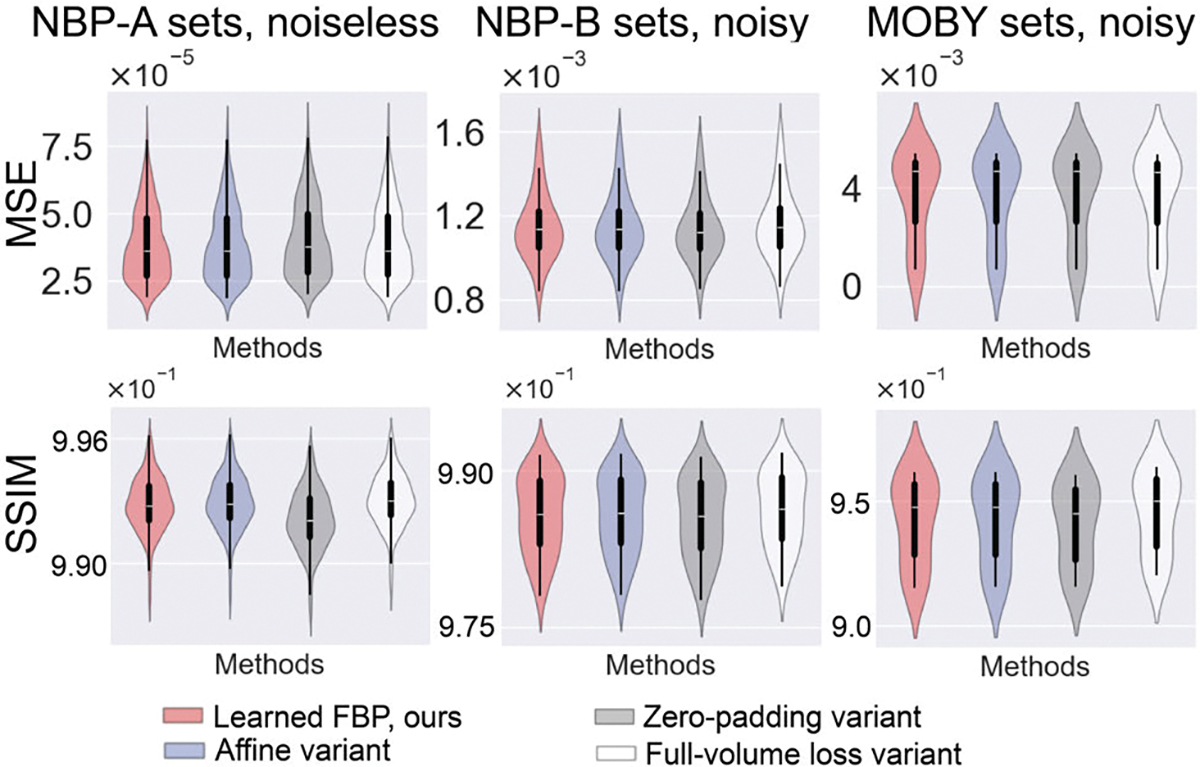
Violin plots of MSE (top row) and SSIM (bottom row) values for the proposed learned half-scan FBP method (red), the affine variant (blue), the zero-padding variant (gray), and the full-volume loss variant (white), on both ID and noisy OOD test sets. Results are presented for 250, 80, and 10 samples from the noiseless NBP-A samples (first column), noisy NBP-B samples (second column), and noisy MOBY samples (third column), respectively. The findings suggest that all models achieve comparable performance on both datasets, indicating that architecture variants and training strategies have minimal trivial impact on reconstruction accuracy.

## Appendix Design Choices in the Model Architecture and Training Strategy

To systematically assess the impact of key design choices in the proposed learned half-scan FBP framework on reconstruction performance, two comparative studies were conducted. The first study investigated linear and nonlinear models for both data-to-data and image-to-image mappings. The second study compared network architecture variations and training strategies. The training, validation, ID test, and OOD test sets for these studies employed the same number of samples as in the main study. However, they were generated from objects with a doubled voxel size (1.0 mm), obtained through spatial downsampling of the objects utilized in the main study. Additionally, the corresponding PACT measurements were generated using a virtual imager configured with only half the number of tomographic views, transducer ring elements, and temporal sampling points. This approach facilitated comprehensive comparisons while maintaining manageable computational demands.

### A. Linear and Nonlinear Models in Data and Image Domains

The proposed learned half-scan FBP method was compared to three alternative DL-based reconstruction methods for half-scan data: a *nonlinear learned half-scan FBP*, a *linear image-to-image U-Net*, and a *nonlinear image-to-image U-Net*. The nonlinear learned half-scan FBP was included to investigate the impact of maintaining linearity in the data-domain filtering operation. The two image-to-image U-Nets were employed to assess the reconstruction performance of learning in the image domain.

The architectures for the linear models excluded activation functions, max-pooling operations, and bias terms, while the nonlinear models incorporated rectified linear unit activations, max-pooling for downsampling, and bias terms in all convolutional layers. The nonlinear learned half-scan FBP model employed the same padding approach, network input, and loss function as the proposed method, as detailed in Section III. For both image-to-image U-Net models, zero padding was applied in all convolutional layers, and the backprojected image $H^{†}p$, obtained from the (unfiltered) half-scan data, was used as the network input. The loss function was defined as the MSE between the network output and the true object. All models shared the identical network depth and number of convolutional channels and were trained using the same random subvolume strategy as described in Section IV.

Figure 7 presents violin plots of MSE and SSIM values for the proposed linear learned half-scan FBP method (red), the nonlinear learned half-scan FBP method (blue), the linear image-to-image U-Net (white), and the nonlinear image-to-image U-Net (gray). Results are reported on both ID (250 noiseless NBP-A samples) and OOD test sets (80 noisy NBP-B, and 10 noisy MOBY samples), respectively. On the ID test set, the nonlinear learned half-scan FBP method performed comparably to the proposed linear learned half-scan FBP. Among all methods, the nonlinear image-to-image U-Net achieved the highest overall performance, while the linear image-to-image U-Net yielded the lowest. However, on the OOD test sets, the proposed method demonstrates superior generalizability, significantly outperforming the other models. These findings suggest that explicitly enforcing linearity in the model supports the stability and fidelity of learning the well-posed linear inverse mapping. Additionally, they underscore the effectiveness of data-domain filtering within the FBP framework, guided by the underlying imaging operator.

### B. Network Architecture Variations and Training Strategies

To analyze the effects of design choices in network architecture and training strategies, an additional study was conducted comparing the proposed learned half-scan FBP method with three variant models. First, to further evaluate the impact of enforcing strict linearity in the network, the proposed method was compared to its *affine variant*, in which all bias terms were included. Second, the effectiveness of the proposed padding approach was examined by comparing it to a model employing standard zero padding, referred to as the *zero-padding variant*. Third, the proposed method was compared with a variant trained using full-volume loss computation, referred to as the *full-volume loss variant*.

Figure 8 presents violin plots of MSE and SSIM values for the learned half-scan FBP method (red), the affine variant (blue), the zero-padding variant (gray), and the full-volume loss variant (white), evaluated on both ID and noisy OOD test sets. To better quantify the performance differences among these variants, Table II reports the mean and the interquartile range (25th to 75th percentile) of these metrics across test sets. All models yielded comparable performance across the datasets. This suggests that once a linear mapping is learned, the inclusion of bias terms (i.e., the affine variant) does not compromise reconstruction accuracy. Although the standard zero padding approach performed similarly to the proposed padding approach, the latter is preferable due to its design aligned with the physical characteristics of the data acquisition process. Moreover, these findings confirm that training with randomly selected subvolumes achieves accuracy equivalent to full-volume supervision, while enabling approximately a 2× speed-up in training time.

**TABLE II:** MSE and SSIM for the Proposed Method and Its Variants on ID and Noisy OOD Test Sets

Method	Learned FBP	Affine variant	Zero-padding variant	Full-volume loss variant
**NBP-A sets, noiseless**				
MSE (×10^−5^)	3.87 (2.70, 4.82)	3.86 (2.70, 4.82)	4.04 (2.82, 4.96)	3.90 (2.72, 4.90)
SSIM (×10^−1^)	9.93 (9.92, 9.94)	9.93 (9.92, 9.94)	9.92 (9.91, 9.93)	9.93 (9.92, 9.94)
**NBP-B sets, noisy**				
MSE (×10^−3^)	1.15 (1.05, 1.22)	1.15 (1.05, 1.22)	1.14 (1.04, 1.21)	1.16 (1.05, 1.23)
SSIM (×10^−1^)	9.86 (9.83, 9.89)	9.86 (9.83, 9.89)	9.85 (9.83, 9.89)	9.86 (9.83, 9.89)
**MOBY sets, noisy**				
MSE (×10^−3^)	3.71 (2.60, 5.04)	3.71 (2.60, 5.04)	3.71 (2.60, 5.04)	3.67 (2.57, 4.98)
SSIM (×10^−1^)	9.42 (9.28, 9.57)	9.42 (9.28, 9.57)	9.40 (9.26, 9.55)	9.45 (9.32, 9.59)

All values are reported as mean (25th percentile, 75th percentile).
